# Method of Material Selection Considering Quality, Environmental, and Cost Aspects

**DOI:** 10.3390/ma18184324

**Published:** 2025-09-16

**Authors:** Andrzej Pacana, Dominika Siwiec

**Affiliations:** Faculty of Mechanical Engineering and Aeronautics, Rzeszow University of Technology, 35-959 Rzeszow, Poland; app@prz.edu.pl

**Keywords:** material selection, material modifications, product quality, life cycle assessment, production engineering, mechanical engineering

## Abstract

Product innovations often involve changes to materials. Various material alternatives are typically sought to improve product quality. Currently, following the current trend of sustainable product development, it is necessary to consider environmental impact. This also applies to materials, particularly their acquisition method. At the same time, modern products should be characterized by low production costs. Despite this, there is a lack of a coherent, standardized method to support decisions about material selection by considering all these aspects during the design or product improvement phase. Therefore, the aim of this research is to develop a method for selecting materials during final product improvement, taking into account quality, environmental impact in the life cycle context, and manufacturing costs. The method is implemented in eight stages and involves calculating the following indicators: (i) quality (satisfaction with use), (ii) environmental impact (for material extraction and acquisition), and (iii) decision-making based on manufacturing costs. Calculations to obtain these indicators were performed using OpenLCA 2.0.0 with the Ecoinvent database, formalized scoring (PS), cost analysis, and morphological analysis. The results of the method lead to the selection of favorable hypothetical prototypes resulting from the proposed material changes. The method was tested using a six-speed manual transmission from a light vehicle. The results showed that the decision-making process for selecting a prototype from among possible material modifications varies. The proposed method can be used for any newly created or modified product at the material selection stage.

## 1. Introduction

Material selection for diverse products is considered one of the most challenging tasks, primarily due to the complex issues involved in multi-criteria decision-making [1,2]. This problem arises in the design of new products and the improvement of existing products that are used in various industrial environments [1]. Materials influence product function, customer satisfaction with product quality, production systems, and the product life cycle assessment (LCA) [3,4,5]. The materials used in a product are selected based on factors such as the customer, usability, costs, and operating environment [6]. For new products, determining the most advantageous material is challenging, especially when considered from a sustainable product development perspective. This depends on the stakeholders and the context in which it will ultimately be used [7,8]. This also applies to production processes, relationships between suppliers and the company, primary resources, recycling options, and energy recovery [9]. It can be assumed that a holistic approach is one that focuses on the selection of materials at an early stage of product development, where the aspects of quality, environmental impact, and production costs are taken into account simultaneously [10,11,12].

Some research has been conducted to support the process of selecting materials for the final product. For example, the authors of [9] developed a framework for sustainable materials selection, taking into account environmental impact. This research was based on a taxonomy and systematization of design approaches. Subsequently, the authors of [13] proposed a method for relating pairs of material procurement and end-of-life product strategies. This method takes into account technical, environmental, and economic factors, including Grey’s relational analysis, supporting the ordering of materials based on several criteria. Another example is [14], which focuses on reducing negative environmental impact during material selection at an early stage of the product life cycle. The focus was on carbon dioxide emissions. The author of [15] conducted a literature review on product sustainability, also considering the stage of selecting materials with the lowest possible negative environmental impact. He highlights the possibilities of complying with the requirements of the ISO 14001 standard [16] and the EMAS regulation, as well as conducting a life cycle environmental impact assessment or eco-efficiency assessment. In addition, the authors of [17] proposed a tool to support decision-makers in selecting materials that meet designers’ requirements. The analysis was based on material quality requirements. The method is based on the assumptions of decision-making methods and is compared during the validation stage, such as, for example, to the TOPSIS method (Technique for Order of Preference by Similarity to Ideal Solution). Furthermore, as the authors of [18] observed, material selection is a multi-criteria decision support problem. The appropriate selection of material for a product is crucial for the success and competitiveness of a company in the global market. Therefore, they proposed the use of two methods, i.e., complex proportional assessment (COPRAS) and mixed data assessment (EVAMIX), for material selection. In terms of material quality analysis, an example is [19], where materials and methods supporting electrochemical sodium synthesis for water purification were verified. In particular, recirculation rates and their impact on the final product were considered. In addition, it is important to mention sustainable materials being developed within the framework of environmental protection. These include biodegradable plastics and biopolymers, as in [20], based on renewable agricultural raw materials and biomass. Biocomposites also have a real chance of replacing glass fiber-reinforced composites. The authors of this article [21] analyzed the application of fibrillated cellulose in materials production. The materials studied ranged from composites and macrofibers to thin films and gels. This type of cellulose is obtained from renewable sources, and when combined with important functional applications, such as mechanical or thermal, it presents technological attractiveness. Sustainable carbon materials are also important, as mentioned by the authors of works such as [22,23]. Various types of carbon materials have already been developed using renewable energy sources, e.g., activated carbons, carbon nanotubes, carbon aerogels, etc., mainly taking into account the entire life cycle.

It was observed that efforts were being made to support the process of selecting materials for products. These studies addressed quality, environmental, and cost aspects. Sometimes, these aspects were combined within a single analysis, but more often, studies focused on qualitative or environmental aspects. Nevertheless, no method was found that would ensure the selection of materials for final products. Existing approaches based on methods such as TOPSIS, COPRAS, or AHP typically do not integrate these three aspects. Furthermore, these methods neglect the selection of materials, which has a significant impact on product innovation. The method presented in this paper not only integrates quality, environmental, and cost aspects but also highlights the important role of materials in creating innovative products. The proposed method can help entrepreneurs and managers offer increasingly higher-quality products to the market while reducing their negative environmental impact, all at a reasonable cost. An additional advantage of the proposed method is the ability to use a separate methodology to evaluate individual aspects (quality, environment, and cost), rather than following a strict methodology. The proposed method would focus on assessing the degree to which customer requirements were met qualitatively (assessing quality in the context of product usability), integrating an environmental impact assessment within the first stage of the life cycle (extraction and sourcing), including linking these results to manufacturing costs. This was identified as a research gap, which the proposed method was intended to address. Therefore, the aim of this article is to develop a method for selecting materials for final product improvement, taking into account quality, environmental impact in the life cycle context, and manufacturing costs.

## 2. Materials and Methods

### 2.1. General Concept of the Method and Procedure

A method for selecting materials for final product improvement was developed, taking into account quality, environmental, and cost aspects. Quality aspects are defined as those that influence customer satisfaction with the product’s quality in the context of its use [24]. Environmental aspects, on the other hand, are defined as those that impact the environment in the context of a product’s life cycle assessment (LCA) [25,26]. Cost aspects are the estimated cost of producing a product. The proposed method will support designers and materials engineers in the initial stages of product design. This will primarily involve the analysis (selection and evaluation) of materials used in the production of the final product. Material analysis focuses on the raw materials obtained and processed in the first phase of the product’s life cycle. This approach applies to the “cradle to grave” approach, where the life cycle phases include (i) material acquisition and extraction, (ii) production, (iii) use, and (iv) end of life [27]. In the proposed method concept, following the authors of [28], it was assumed that materials selection can be implemented according to two approaches: (i) design of a new product, and (ii) modification of an existing product.

In accordance with the adopted approach, based on the assumptions of the materials selection approach proposed in [28], a framework for materials selection for modified final products was developed (taking into account quality and environmental aspects). The method was developed in eight main stages:

1. Selecting a product for analysis;
2. Identifying product criteria (quality characteristics);
3. Developing a shortlist of materials and their alternatives for key product criteria;
4. Evaluating the quality of components for proposed material modifications;
5. Assessing the environmental impact of material modifications;
6. Estimating the quality-environmental (QE) index;
7. Quality-environmental-cost (QEC) analysis;
8. Selecting the most favorable material alternatives based on product prototypes.

**Figure 1:**
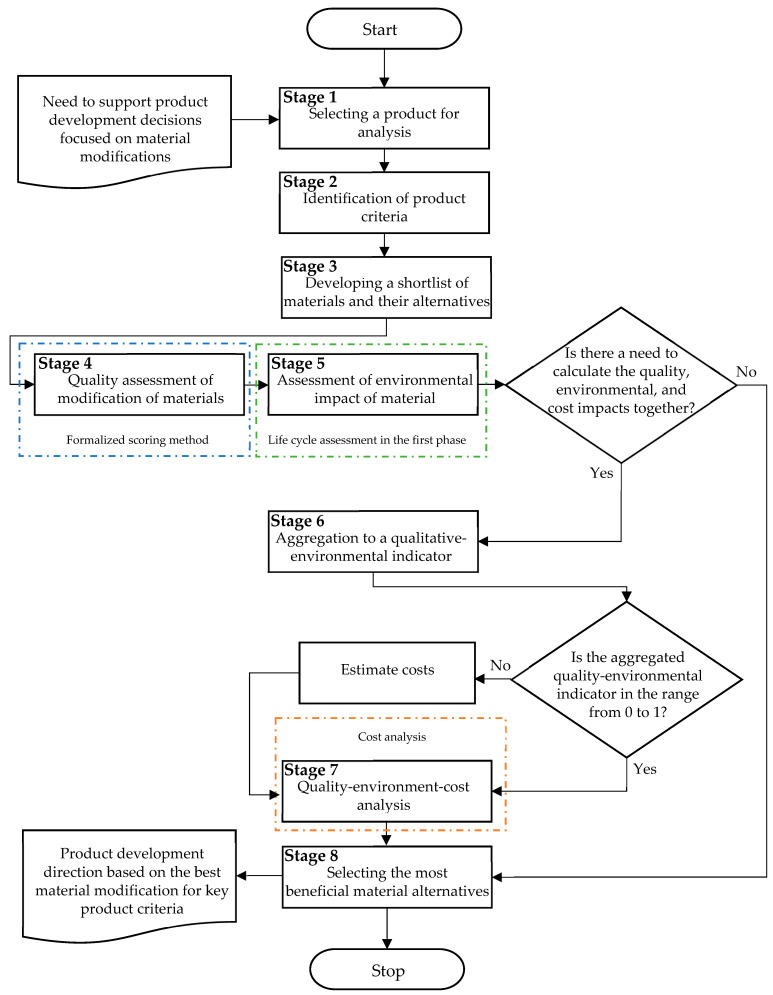
Scheme of analysis of material alternatives in terms of quality, environment, and cost.

Taking these steps into account, a method flowchart was developed, which is presented in Figure 1.

The proposed method is designed for significant modification of existing products, often related to quality assessment, cost optimization, and environmental protection. This approach is consistent with sustainable product development and is thus adapted to the dynamically changing needs of the market, which requires the creation of ever-newer (innovative) products [29].

### 2.2. Main Assumptions of Method

To generalize the method with the goal of applying it to various cases (industries or other products), key assumptions were adopted. These were based on the authors’ previous research, including the state of the art from a literature review. The basic assumptions for the method are as follows:

- The minimum number of key product quality criteria (features, attributes) is three (those that directly impact customer satisfaction with product use, including those requiring modification) [30];
- At least three alternative states, i.e., modifications to the materials of key product criteria, are developed, but no more than nine states per criterion [31];
- During material changes, the quality of the product, module, or subassembly will not be lower than the current one;
- The environmental impact of material modifications is assessed in the context of the life cycle, limited to the first stage (material acquisition and extraction).

The rationale for the adopted assumptions is presented during the presentation of the method stages, including an in-depth literature review, as in Section 2.3.

### 2.3. Comprehensive Presentation of the Method and Justification of the Adopted Assumptions

The method is presented according to eight main stages. The characterization provides a more detailed justification of the assumptions made, supported by a literature review.

**Stage 1. Selecting a Product for Analysis**

The first stage involves selecting a product for testing. Any product can be analyzed. Decisions in this regard are made by the entity using the proposed method, e.g., the product manager [32]. The product to be analyzed may have specific specifications (e.g., machine components), but in this respect, there is often a relatively small degree of modification, usually resulting from the expected product quality (parameters, e.g., strength). Therefore, the method is primarily dedicated to products commonly used by customers, understood as the overall (final) product. In such cases, product selection may depend on the dynamics of changing customer requirements, the need for product development due to emerging product innovations, or even when the product reaches market maturity.

**Stage 2. Identification of Product Criteria (Quality Features)**

The second stage is the identification of product criteria. Product criteria are defined as quality features (attributes), meaning those that directly impact customer satisfaction with the product’s usability. Therefore, these criteria primarily concern performance, functionality, and even visual characteristics, such as power, length, thermal conductivity, weight, color, etc. These criteria vary depending on the product being analyzed. They are selected by a team of experts, such as a designer, technologist, quality engineer, and others. The team may select these criteria during brainstorming (BM) [33], including based on catalogs (specifications). Depending on the product’s complexity, the number of criteria may vary. For example, a moderately complex product typically has 10–15 criteria [34]. It is assumed that the team of experts identifies only key product criteria, meaning those requiring improvement (modification). According to the authors of the method, at least three key criteria should be identified for further analysis, an assumption supported by previous research, e.g., ref. [30].

**Stage 3. Developing a Shortlist of Materials and Their Alternatives for Key Product Criteria**

The third step of the method involves identifying materials that could potentially be considered as alternatives to the product criteria requiring improvement. This involves proposing various material modifications for the key product criteria (from step 2 of the method). Material selection is typically performed by designers and materials engineers. This is a crucial step, determining not only the quality of the final product but also the production efficiency, user satisfaction, and possibility of its potential recycling after use [35].

Basic materials are raw materials, which are used as inputs to the production of products in various industries. According to industrial production research conducted by the Polish Statistical Office [36], a raw material, within product research, is an unprocessed material of animal, plant, or mineral origin, or resulting from waste processing, which can be used as a component in the production of final products, energy, or intermediate materials used in the production of new products. A raw material is a material substance that is created unprocessed by humans in the natural environment, including extraction as a result of human activity [37]. This means that extraction of the raw material has already taken place, and natural matter itself, undisturbed in the environment, is not yet considered a raw material. Therefore, it is assumed that after the materials are obtained, metals and minerals, including agricultural products and synthetic compounds, are processed into a finished (final) product. Raw materials include minerals, but also other natural resources from mines, including products manufactured from these raw materials, as well as those requiring further processing. It is assumed that the production process generates a finished product or a semi-finished product from the obtained raw materials [38].

The importance of precise selection and proper processing of these materials determines economic growth, including the creation of innovation. Therefore, a proper understanding of the properties of raw materials (primary materials) is crucial for producers, engineers, and researchers, as these raw materials have a direct impact on production processes, cost effectiveness, and the commonly understood quality of final products [38].

The approach to selecting and evaluating materials (in terms of raw materials) can be considered in the context of the product life cycle. In this case, the acquisition and processing of materials constitute the first phase of the life cycle, with subsequent phases including production, use, and end of life. This is a “cradle-to-grave” approach to product design [39,40]. A literature review revealed that selecting materials with additional considerations of quality and cost aspects is problematic. A solution to this problem was proposed by proposing a method based on universal criteria for assessing the quality and environmental performance of materials in the first phase of the product life cycle. These criteria were developed based on a literature review and the OpenLCA 2.0.0. program with Ecoinvent databases from environmental assessment programs. The focus was on industrial raw materials due to their widespread use in consumer products.

The proposed method assumes that a raw material is a natural resource that has not been processed but will be used in the production of industrial products. The quality and properties of raw materials have a direct impact on the efficiency of the production process and especially on the quality of the final product [38,41].

Raw materials are classified as primary and secondary, and these may be repeated in the processing cycle, as some materials can be reused. Other classifications also exist, as indicated by the authors of studies [38,41]. Raw materials in industry are classified according to the following categories:

- mineral resources—extracted from beneath the earth’s surface, e.g., metal ores (minerals such as iron, copper, diamonds), energy sources (e.g., crude oil, natural gas, coal);
- agricultural resources—cultivated in agriculture and used in industry, e.g., cereals (e.g., wheat, oats), vegetable oils (e.g., cottonseed oil, soybean oil);
- natural resources—naturally occurring materials, e.g., firewood (e.g., used in construction and the furniture industry), salt (e.g., used in the food and chemical industries);
- artificial resources—created through chemical reactions, e.g., plastics (e.g., polyethylene, polypropylene, PVC), synthetic fibers (e.g., nylon, polyester).

However, according to [38,42], it is possible to adopt the classification of raw materials as:

- natural resources—extracted or harvested directly from the earth, e.g., mineral resources (e.g., iron ore and bauxite), fossil fuels (e.g., coal, crude oil, natural gas), agricultural products (e.g., wheat, cotton, timber);
- processed materials—obtained from natural resources, e.g., steel (from iron ore), plastics (from petrochemicals), cement (from limestone);
- recycled materials—recovered and processed for reuse, e.g., paper, metals, plastics, construction waste (e.g., rubble, concrete waste), hazardous waste (e.g., electronics, asbestos), organic waste (e.g., food waste).

Unprocessed or minimally processed raw materials are also observed and used in the production of products. As reported by [38], they are classified into two main categories:

- biological materials—agricultural products, such as cotton, wood, and food, which come from nature and are renewable;
- mineral and synthetic materials—metals, such as iron and aluminum, and non-metals, such as silica and polymers, which are extracted from the earth or synthesized through chemical processes.

Subsequently, it is possible to distinguish the following groups of materials: metals, ceramics, polymers, and composites. This division is visible in engineering sciences, primarily in the process of selecting materials for product design. This classification is based on the dominant bond type and, therefore, can be applied to the integration of material substances, as in [37]. However, taking into account the assumptions of the authors of the book [43], materials can be classified according to their multiplicity and visual representation. This approach integrates aesthetic and perceived attributes, which favors the design of products where visual impact is important. It has been observed that material classifications can depend on measured properties, leading to the specificity of the final product in which the material raw materials will be incorporated [37]. These properties include, for example, hardness, strength, elasticity, plasticity, ductility, thermal and electrical conductivity, and biodegradability [44,45].

**Figure 2:**
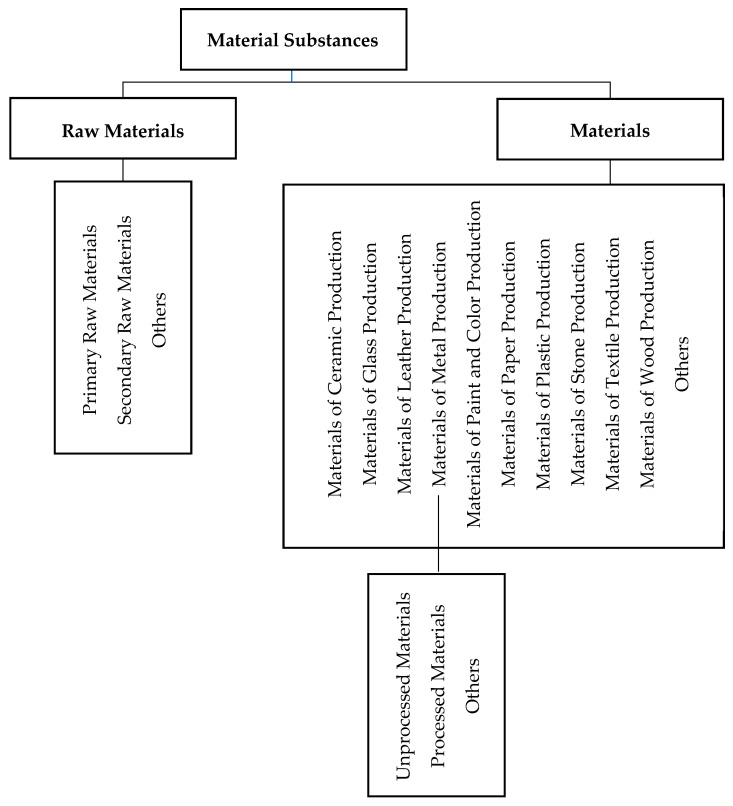
Classification of materials. Own elaboration based on [37].

According to these assumptions, the classification of materials was developed by the authors of [37], as shown in Figure 2.

In addition, according to [46], material classifications may include, among others, textiles/leather, metal, plastic, composites, elastomer/rubber, wood, ceramics/stone, glass, etc. In summary, based on the literature review, the aforementioned material classifications were compiled, as shown in Table 1.

When analyzing the available groups (classifications) of materials presented, for example, in [37,38,41,43,47,48], they are divided into so-called material classes. Therefore, based on an analysis of the relevant literature, the authors propose classifying materials into the following classes:

- raw (primary) materials, e.g., metals (e.g., steel, aluminum, copper), glass, wood, stone, and minerals (e.g., concrete, cement, plaster), paper, and cardboard;
- organic and biodegradable materials, e.g., wood and wood-based products, natural fibers (e.g., cotton, linen, wool), bioplastics (e.g., polylactic acid), compostable plastics;
- energy (fuel) materials, e.g., fossil fuels (e.g., coal, crude oil, natural gas), biofuels (e.g., bioethanol, biodiesel), renewable energy (e.g., firewood, pellets);
- auxiliary and consumable materials, e.g., chemicals (e.g., paints, adhesives, solvents), lubricants and oils, industrial gases (e.g., nitrogen, argon);
- composite materials, e.g., polymer composites (e.g., carbon fiber in an epoxy matrix), composites, metals, laminates;
- waste materials (for disposal or recycling), construction waste (e.g., rubble, concrete waste), hazardous waste (e.g., electronics, asbestos), organic waste (e.g., food waste).

**Figure 3:**
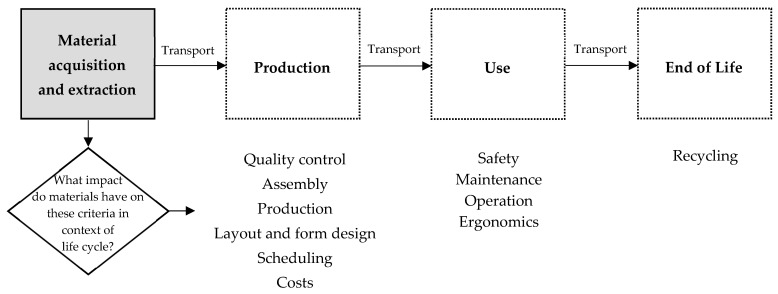
Examples of knowledge factors related to material selection in the context of the product life cycle. Own elaboration based on [50].

Designers tend to use individual methods when selecting materials. This stems from the fact that materials influence various aspects of product design, such as form, function, and production technology, as well as sensory experiences and evoked emotions [47]. There are no rules that explicitly consider qualitative, environmental, and cost aspects simultaneously when selecting materials. At the same time, no single, common, systematic approach has been found to support designers in the material selection process during product design [47]. It is assumed that materials are selected by a team of experts and technologists [49]. The team selects the most advantageous materials from among those available in a given case, guided by established selection criteria, such as material properties, functionality of the final product, and economic conditions. The more complex the final product, the greater the number of materials, and the more difficult their selection becomes. Following the authors of [50], when selecting materials, it is possible to be guided by specific factors that are included in the product life cycle, as shown in Figure 3.

According to [35], material selection often comes down to considering various design constraints, such as properties, function, process, and shape. When identifying the most advantageous materials, it is also necessary to analyze specific material properties. Following [1], it is assumed that many materials are excluded from the selection process, and only a small number of materials are considered potential candidates for the materialization of designed products. At the same time, it is important to pay attention to the intended design goals, including the need to care for the environment, which, in this case, manifests itself through so-called green materials [1]. This is a difficult task, especially as materials selection resources are developing, as are databases and libraries of physical materials, but also computer programs. At the same time, material selection should be carried out taking into account stakeholder behavior and industry requirements, including compliance with regulations and other issues [2]. Therefore, when selecting materials, it is essential to consider all issues in the context of the product life cycle, including striving for cost reduction and meeting product performance requirements. The selection of materials can be supported by selected MCDM (Multi-Criteria Decision Methods) techniques [51], but also by, e.g., an expert system [52,53], the Pugh method [54], or software, e.g., the Cambridge Engineering Selector (CES) [35] or databases of environmental assessment programs containing libraries of a set of different materials, e.g., OpenLCA [55].

Finally, a short list of materials is developed that can be alternatives to the current product criteria materials. These materials are selected for the product criteria (features) requiring change, and selecting the appropriate one often poses a decision-making challenge. Therefore, it seems necessary to seek a universal method to support these decisions, if possible. As part of the process of finding the most advantageous material, no more than 7 ± 2 material alternatives should be identified for a single criterion (feature) [31]. It is assumed that the material change (product, module, or subassembly quality) will not be lower than the current one. The materials selected for analysis are evaluated in the next stage of the method development.

**Stage 4. Quality Assessment of Elements for Modification of Criteria Materials**

In the fourth stage of the method, the qualitative assessment of the material modifications to the product criteria is performed. This means that the proposed material modifications will be evaluated in terms of meeting customer expectations (satisfaction with use). The analysis is performed by a team of experts, e.g., a designer, technologist, or quality engineer. The proposed method assumes the use of formalized scoring (PS) [34]. This is a simplified method for estimating product quality, also known as the Czechowski method [56]. The method is based on ratings on a five-point Likert scale. Therefore, initially, all proposed material modifications to the product criteria are rated on this scale, where 1—very unfavorable modification, 2—unfavorable modification, 3—intermediate modification, 4—favorable modification, and 5—very favorable modification. Based on these ratings, the quality level of the product prototype materials is assessed using the formalized scoring methodology. The basic formula for estimating the quality level of material modification of a given prototype is (1) [34,56]:

(1) $$Q_{i}=G_{i}+K_{i}-C$$

where Q—quality index of material modification of the i-th prototype, G—main term for the i-th prototype, K—correction term regulating the influence of undesirable states for the i-th prototype, C—constant (0.05 for standard requirements, 0.01 for stricter requirements), and i = 1, 2, …, n.

The main term (G) is calculated from Formula (2) [34,56]:

(2) $$\left\{\begin{array}{c} G_{i}=\frac{P_{i}}{8\cdot n}\\ P_{i}=\left(9\cdot a_{i}+7\cdot b_{i}+4\cdot c_{i}+2\cdot d_{i}+e_{i}-n\right) \end{array}\right.$$

where P—polynomial of the scoring result of the i-th prototype, taking into account the importance coefficients of the assessments of the states (modifications) of the material criteria; n—number of considered modifications of the criteria; a, b c, d, e—number of assessments for the i-th prototype, respectively, with 5, 4, 3, 2, and 1 points awarded to a given modification of the criterion; and i = 1, 2, …, n.

The correction term (K) regulating the influence of undesirable states (modifications) should be calculated from Formula (3) [34,56]:

(3) $$K_{i}=\frac{c_{i}+5\cdot d_{i}+10\cdot e_{i}}{200\cdot n}$$

where K—correction term of the i-th prototype regulating the influence of undesirable material modifications in a given prototype criterion; n—number of considered material modifications for the prototype criterion; c, d, e—number of assessments for the i-th prototype of 3, 2, and 1 points, respectively, awarded to a given material modification of the prototype criterion; and i = 1, 2, …, n.

Based on the estimated quality index of materials selected for the key product criteria (Q), it is possible to determine the degree of customer satisfaction, i.e., user satisfaction. The Q index value should range from 0 to 1, where 0 indicates a lack of customer expectations (quality requirements) met, including minimizing customer satisfaction during product use, and 1 indicates full customer expectations (quality requirements) met, including maximizing customer satisfaction during product use. Based on the Q index, a ranking of prototypes can be developed. The first position is taken by the prototype with the highest quality index, indicating that this prototype has the expected material modifications for key product criteria. The analysis is supplemented with the environmental aspect in the next step of the method.

**Stage 5. Assessment of the Environmental Impact of Modifications to Product Criteria Materials**

The fifth stage of the method involves assessing the environmental impact of material modifications to prototype criteria. A literature review revealed that environmental impacts are increasingly important in modified products [57,58,59]. Considering that this is also crucial for sustainable product development [60]. The proposed method assesses the environmental impact of material modifications in the context of the first stage of the product life cycle (LCA). This encompasses the “cradle to grave” approach, i.e., material sourcing and extraction, production, use, and end of life. Therefore, in accordance with the LCA method presented in the ISO 14040 standard [61,62], it is additionally proposed to adopt a functional unit to ensure data normalization. The method concept is based on assessing the environmental impact of the materials used in the product (specifically, in a selected criterion/attribute of the product). Therefore, it is proposed that the functional unit is calculated depending on the weight (mass) of the final product. For example, the final product is a manual six-speed passenger car gearbox. The weight of one such gearbox is approximately 50 kg. According to the proposed assumptions, it constitutes a functional unit in a given analysis. Because the proposed method only covers the environmental assessment of various material alternatives, the system boundaries apply to the first stage of the LCA (i.e., material sourcing and extraction). It is recommended to assess the environmental impact based on the selected environmental burdens required for a given type of analysis. For example, a popular example is the carbon footprint, which estimates total greenhouse gas emissions. These are expressed as carbon dioxide (CO_2_) equivalents. Computer software such as OpenLCA [55] is helpful in assessing the environmental impact of materials from given prototypes. This yields the so-called environmental impact index (e). After calculating the environmental impact of the prototypes, it is recommended to compile them into a single ranking. The higher the environmental burden value (e), the less favorable the modification of the material criteria. The results of the qualitative assessment of material modifications are combined with the environmental assessment in the next stage of the method.

**Stage 6. Estimation of the Quality–Environmental Index (QE)**

The sixth step of the method involves integrating the qualitative assessment results with the environmental impact assessment in the context of the product’s material modifications. This creates a quality-environmental (QE) index, which provides a simultaneous interpretation of the qualitative and environmental performance of the prototypes offered. The index after the qualitative assessment (according to the PS method) has values from 0 to 1, where higher values mean better. In turn, the index after the environmental impact assessment can take values significantly above 1 (without the possibility of specifying a precise range), where higher values mean worse. Therefore, it is initially necessary to normalize the environmental index so that it takes values from 0 to 1. Only then will it be possible to compare it with the qualitative index and integrate them. Normalization of the environmental index is performed according to Formula (4):

(4) $$E_{i}=\frac{\max e_{i}-e_{i}}{\max e_{i}-\min e_{i}}$$

where e—indicator of the impact of product material modification on the environment in the context of the life cycle, i—prototype, and i = 1, 2, …, n.

This relationship allows for the environmental indicator values to be normalized to a range of 0 to 1. Simultaneously, the original indicator values are inverted to be consistent with the principle—the more, the better, and the less, the worse (as in the case of a qualitative indicator). Furthermore, it is possible to aggregate the qualitative indicator with the normalized environmental impact indicator (QE), as in Formula (5):

(5) $${QE}_{i}=\frac{Q_{i}+E_{i}}{2}$$

where Q—quality index of material modification of the i-th prototype, E—normalized environmental index of material modification of the i-th prototype, and i = 1, 2, …, n.

The quality–environmental (QE) index for material modifications of prototypes ranges from 0 to 1. This index allows for the creation of a further prototype ranking. Its interpretation is as follows: the higher the index, the more favorable the material modification is in terms of meeting customer requirements (quality), while simultaneously having a low negative impact on the environment over its life cycle. The cost effectiveness of these solutions is verified in the next step of the method.

**Stage 7. Quality–Environment–Cost Analysis (QEC)**

The seventh step of the method involves conducting a cost analysis. The goal is to analyze whether the proposed prototypes will be cost-effective. In the proposed method, this involves aggregating the quality–environmental indicator with the actual production cost of the prototypes. This allows for the simultaneous analysis of the prototypes’ quality, environmental, and cost performance. Aggregation of the indicators with the actual production cost is possible through the use of a cost analysis, in which the cost–dependency decision function is presented as (6):

(6) $$D=f\left(K,QE\right)$$

where: K—prototype cost and QE—value of the quality–environmental indicator.

It is necessary to estimate the cost index ($C_{K}$), the relative cost (k), the cost proportionality index (E), the decision function index (d), and the cost index (c) (7):

(7) $$\left\{\begin{array}{c} C_{K}=\frac{K}{QE}\\ k=\frac{K_{a}-K}{K_{a}-K_{i}}\\ \begin{array}{c} E=\frac{k}{QE}\\ \begin{array}{c} d_{0}=0.5\cdot E\\ \begin{array}{c} d_{1}=0.5+0.5\cdot \left(1-\frac{1}{E}\right)\\ c=\frac{C_{Ka}-C_{K}}{C_{Ka}-C_{Ki}}\; \end{array} \end{array} \end{array} \end{array}\right.$$

where K—estimated cost of the prototype, QE—quality–environmental indicator for C_k_ expressed as a percentage or for E expressed as a decimal fraction, k—relative cost (relative cost intensity in the area of a given variability), K_a_—the highest cost in a given analysis, K_i_—the lowest cost in a given analysis, d_0_—decision function index estimated for E ϵ $\langle 0;1\rangle$, d_1_—decision function index estimated for E > 1, where E—cost proportionality index, $c_{Ka}$—maximum relative cost index in a given analysis, $c_{Ki}$—minimum relative cost index in a given analysis, and $c_{K}$—maximum relative cost index in a given analysis.

The calculations are performed separately for all analyzed prototypes. The estimated decision-making indices for the technical (R_t_) and economic (R_e_) preferences are assumed, as well as the average decision-making indices (R_d_) (8):

(8) $$\left\{\begin{array}{c} R_{t}=\frac{\alpha QE+\beta d+\gamma c+\delta k}{\alpha +\beta +\gamma +\delta }\\ \alpha :\beta :\gamma :\delta =8:4:2:1\\ \begin{array}{c} R_{t}=0.0667\left(8QE+4d+2c+k\right)\\ \begin{array}{c} R_{e}=\frac{\alpha k+\beta c+\gamma d+\delta QE}{\alpha +\beta +\gamma +\delta }\\ \begin{array}{c} R_{e}=0.0067\left(8k+4c+2d+QE\right)\\ QEC=0.5\left(R_{t}+R_{e}\right) \end{array} \end{array} \end{array} \end{array}\right.$$

where QE, k, d, c, k—as in Formula (7) and α:β:γ:δ—importance indicators.

The decision-making indicator (QEC—quality–environment–cost) should have a value between 0 and 1. If this indicator falls outside the specified range, the calculations should be repeated until the desired result is achieved. QEC ensures decision-making about the direction of product development, as represented by the next stage of the model.

**Stage 8. Selection of the Most Advantageous Material Alternatives According to Product Prototypes**

The eighth and final step of the proposed method is to select the prototype with the most favorable quality, environmental, and cost performance indicators. In this case, it involves selecting the most favorable material alternatives presented by the product prototypes. Decisions in this regard are made based on the QEC index (from step 7). Prototypes should be ranked according to this index, with the higher the value, the better. The first position in the ranking is the prototype with the highest (maximum) QEC value. The last position in the ranking is the prototype with the lowest (minimum) QEC value. The QEC index can be interpreted according to a scale of relative states, as in Table 2.

According to the method’s concept, it is recommended to select the prototype with the highest QEC score. It is characterized by (i) the expected quality of customer requirements, (ii) the relatively lowest negative environmental impact in the life cycle context, and (iii) the cost-effectiveness. Nevertheless, the final decision regarding prototype selection rests with the expert conducting the analysis. This choice may also depend on other individual preferences, such as resource constraints.

The article was tested using a six-speed gearbox for light motor vehicles as an example. This product was presented in studies such as [63,64,65,66,67].

## 3. Results

The test and illustration of the method were conducted using a manual six-speed gearbox for a light motor vehicle. The choice of this product was determined not only by its universality but also by the regular improvement efforts undertaken as a result of technological progress in the automotive industry. This creates a need for continuous improvement in terms of gearbox durability, efficiency, and comfort.

Following the steps of the proposed method, basic gearbox criteria were determined. These criteria relate to the basic usability and performance functions of this product. They were selected based on publicly available gearbox catalogs, including the housing, gears, synchronizers, bearings, catches, seals, and shafts. This is a relatively simple product, consisting of seven main criteria. Because each of these components can have many different material modifications, three key criteria were selected: the housing, gear, and synchronizer ring. This method met the assumptions regarding the minimum number of three key criteria for analysis. The gearbox housing provides external protection for the internal components, such as shafts, gears, and gear mechanisms. It is an integral part of the product, ensuring its proper functioning. The next component, the gear, is part of the gear shift mechanism. It ensures power transmission from the engine to the vehicle wheels, thereby ensuring speed and torque changes. The synchronizer, on the other hand, allows for smooth gear changes. It equalizes the rotational speed of the shafts and gears. This prevents jerking and grinding during gear changes. The main element of the synchronizer is the ring. Further steps of the method were tested based on the analysis of the three components (criteria) of the gearbox: the housing, the gear (generally five or six pairs), and the synchronizer ring.

Next, a short list of materials for key gearbox criteria was developed. Based on expert knowledge and experience, including publicly available product catalogs, the following basic materials were proposed:

- housing: aluminum, cast iron, magnesium alloy, or reinforced composites;
- gear: aluminum, cast iron, bronze, plastic, sintered metals;
- synchronizer ring: bimetal (brass–bronze), chrome steel.

Based on these, five different material alternatives were created for the analyzed gearbox criteria. An example set of material alternatives for a manual gearbox for the three key criteria is presented in Table 3. The material alternatives were developed based on, among other factors, the main benefits and limitations that determined the presented material combinations for the selected gearbox criteria.

Next, the proposed modifications to the gearbox criteria materials were assessed. The assessment was conducted qualitatively, i.e., in terms of meeting customer expectations (satisfaction with use). A formal scoring method was used. Initially, each material modification was assessed on a Likert scale, and the results are presented in Table 4.

Next, using Formulas (1)–(3), calculations were made according to the PS method. The partial results and the Q index, along with the prototype ranking, are presented in Table 5.

A qualitative assessment of the modifications to the gearbox’s material criteria revealed that the P5 prototype was the most advantageous. It had a significantly higher quality index (0.70) compared to the other prototypes. The P4 prototype performed the worst (0.45). This means that the highest degree of customer satisfaction (satisfaction with use) can be achieved with material modifications, including an aluminum housing, steel gears, and a copper–bronze (brass–manganese) synchronizer.

**Figure 4:**
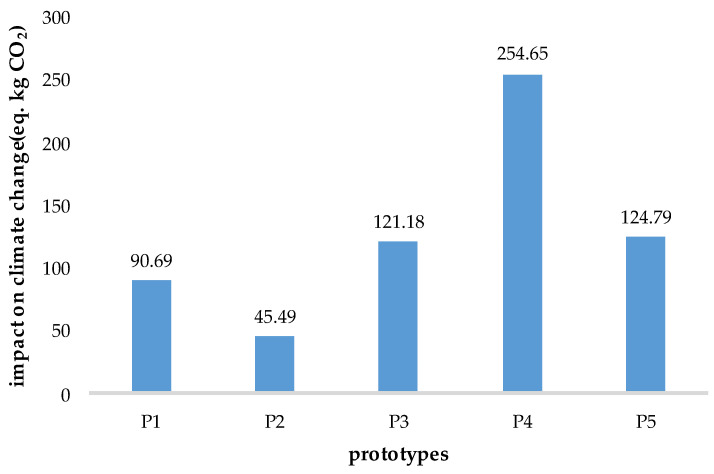
Result of the assessment of material modifications of gearbox prototypes in terms of environmental impact for the climate change criterion per kilogram of carbon dioxide equivalent.

Next, an environmental impact assessment of the proposed material modifications to the gearbox was conducted. This was performed as the first stage of the life cycle assessment (material acquisition and extraction) using a “cradle-to-grave” approach. This represents the system’s boundaries. Within these boundaries, five gearbox prototypes, differentiated by material modifications, will be considered. The functional unit for data normalization is the average weight of one gearbox unit. This was assumed to be 50 kg. Based on this functional unit, the average weights of the analyzed criteria were estimated for the analysis. This was based on product catalogs, such as Hanlon Motorsports. The gearbox housing was assumed to weigh 10 kg. A single gear wheel weighs approximately 1.5 kg to 3.5 kg. The analysis assumed that six basic gear wheels would be included, one with an average weight of 2 kg. Therefore, the total weight of the gear wheels in the analyzed gearbox could be approximately 12 kg. The weight of the gearbox synchronizer ring was also estimated. The entire assembly—hub, bushing, and rings—weighs from approximately 0.45 kg to 2.3 kg for a typical six-speed manual transmission. Six sets of synchronizer rings are considered, with an average weight of 1 kg each. The total weight of the synchronizer rings is, then, 6 kg. Based on these assumptions, calculations were made of the environmental impact of material modifications to the gearbox. The environmental assessment was conducted using OpenLCA 2.0 with the Ecoinvent database. The analysis of material modifications was performed for the environmental criterion of climate change (according to the CML v4.8 impact analysis), which is the verified environmental burden. This criterion also takes into account carbon dioxide emissions, communal metals, and tetrafluoromethane. The unit is the kilogram of CO_2_ equivalent. Equivalent CO_2_ was adopted because it is a popular indicator of environmental impact. Of course, other indicators exist, and the choice of a specific one depends on the specificity of the product being analyzed. Considering the variations in materials, CO_2_ seemed to be the most advantageous choice for testing the method. The results are presented in Figure 4.

According to the adopted assumptions, prototype P2 has the smallest negative impact on climate change (45.49 kg eq. CO_2_). Considering only the environmental aspect, it would prove to be the most beneficial, i.e., environmentally friendly. Prototype P1 demonstrated a relatively small negative impact on climate change (90.69 kg eq. CO_2_). In turn, prototype P4 has the largest negative impact on climate change (254.65 kg eq. CO_2_). This prototype also proved to be the least desirable in terms of quality, where it also ranked last. Prototype P5, with the highest position in the qualitative ranking, ranks fourth in the environmental analysis, with an environmental burden for climate change of 124.79 kg eq. CO_2_.

Therefore, certain differences are observed between the results of qualitative and environmental analyses of material modifications for selected gearbox components. At this stage, the choice of prototype would depend on the importance of one aspect over another for the entity conducting the analysis, e.g., a designer, technologist, or manager. However, the method’s concept assumes the aggregation of qualitative and environmental aspects and, then, supplementing the analysis with the cost aspect. As stated in the proposed method, it is necessary to normalize the environmental indicator to a value on a scale of zero to one, including a reverse ranking, where the higher the value, the better. Formula (4) is used for this purpose. Subsequently, if these aspects (quality and environmental) are assumed to be equivalent, Formula (5) is applied, aggregating them into a single decision-making indicator (QE). The results are presented in Table 6.

By simultaneously considering quality and environmental aspects, we determined that prototype P2 was the most advantageous in this comparison. It simultaneously meets customer requirements regarding product use and is also relatively environmentally friendly. A broader interpretation of the results is performed in the final stage of the method.

Next, a quality–environmental–cost analysis was conducted, in which the estimated cost of the prototypes under consideration and the previously calculated QE index were entered. The proposed method was followed (as explained in detail in stage 7). Formulas (6)–(8) were applied. The analysis results are presented in Table 7.

Taking cost into account, along with the aggregated quality–environmental indicator, resulted in changes in the prototype rankings obtained in the previous stages of the method. In this case, prototype P1 proved to be the most advantageous. It demonstrates the expected modification of the gearbox criteria materials. This modification simultaneously meets quality, environmental, and cost requirements. Prototype P2 is the least advantageous after taking cost into account. Considering all three aspects simultaneously, it can definitely be rejected, as confirmed by the negligible QEC index.

**Figure 5:**
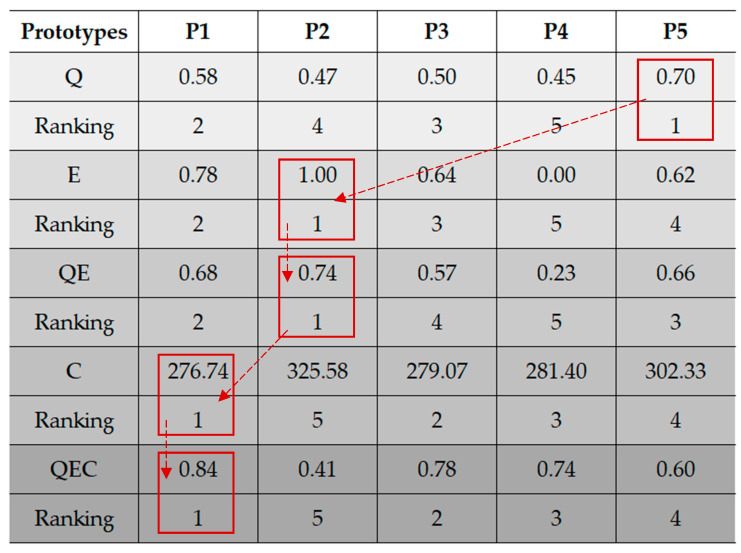
Morphological analysis of the method results.

A comprehensive interpretation of the results and selection of the most favorable prototype occurs in the final stage of the method. Therefore, all obtained method parameters and gearbox prototypes (material modifications of the selected criteria) were compared. The results of the method are presented in a morphological analysis (Figure 5).

In the qualitative analysis, prototype P5 is the most favorable (0.70). It has a significant advantage over the others. In turn, considering the environmental aspect, this prototype took fourth place (0.62). A change in rankings occurred with prototype P2, which proved to have the least negative environmental impact. However, considering both the qualitative and environmental aspects, some changes in the ranking of material modifications are observed. P2 remains the most favorable prototype (0.74). This is due to relatively small differences between the indicator values for the qualitative aspect. This applies to prototypes P1 and P4, where the range of changes is within 0.13 of the difference in these values. In this case, the environmental aspect has a significant impact, with a clear discrepancy between the indicator values for the considered prototypes. Next, the estimated cost of the prototypes was verified. Prototype P1 had the lowest cost value. Prototype P3 is next, and prototype P2 is the most expensive. After considering costs, quality, and environmental aspects simultaneously, it was the P1 prototype that best met all these criteria. It ranked first in the ranking (0.84). On a relative scale, choosing this prototype was a distinctive decision. This prototype had always ranked second in previous rankings. This means it was rational to choose it as the prototype for the development of the analyzed gearbox.

## 4. Discussion

Product design and improvement are increasingly focused on sustainability. All stages play a crucial role in this process, the first of which is the selection of materials for the product. Material selection is based, among other things, on specific properties and the company’s available resources. This is a multidisciplinary task, requiring the interaction of multiple stakeholders, such as product designers, material scientists, engineers, and even customers and end users. The challenges encountered during material selection are typically complex, often based on decision-makers’ intuition. However, design should be more systematic and rigorous, which is achieved by considering not only quality requirements but also environmental impact and costs.

The aim of the research was to develop a method for selecting materials for final product improvement, taking into account quality, environmental impact in the life cycle context, and manufacturing costs. The method was developed in eight main stages and tested using a six-speed manual transmission used in light vehicles. After testing the method, the main benefits of the method were observed, i.e.,:

- support for designers, engineers, and technologists, for example, in the decision-making process when designing new products or improving products already on the market;
- selection of product materials based on: (i) the quality aspect, directing the improvement process towards meeting customer requirements (increased product satisfaction); (ii) the environmental aspect, analyzing the environmental impact of materials in the context of the first stage of the product life cycle; and (iii) the cost aspect, relative to the cost of production (manufacturing);
- selection of product materials according to the concept of sustainable development, where all key aspects (quality, environmental, and cost) are important;
- the method is relatively inexpensive and time-efficient, providing a solution to a multi-criteria decision problem by analyzing several commercially available materials for various product criteria in the form of preliminary design alternatives;
- support for the analysis with other techniques, such as the formal scoring method, OpenLCA, cost analysis, and morphological analysis.

The method also has limitations, such as the need for software for environmental assessment of materials, the inclusion of only selected product criteria, the lack of analysis of all possible material combinations for a product, and the need for an interdisciplinary team of experts with expertise in quality, environmental, and cost. Another limitation is the human factor, but it exists in every decision-making problem in production processes. It is worth noting that the proposed methodology utilizes teamwork, so the results are a kind of averaging of the opinions of the team’s experts. This is a much safer solution than, for example, agile development (Agile), where much often depends on a single expert. Subjectivity in the proposed method is reduced by the skillful selection of team members, ensuring that their knowledge and skills complement each other. Also, the presented method covers the material analyses considered in the first stage of life cycle assessment, i.e., material sourcing and extraction. The method does not include other stages, such as recycling, but future research will expand upon the method. The method’s increased time consumption may be noticeable during initial attempts, when it is not yet well-known. However, after testing the method and becoming familiar with its procedure, the time required for subsequent use can be significantly reduced. This requires the involvement of a competent team of experts responsible for its implementation.

In the near future, it would be beneficial to develop a simple application that will be more complex, for example, with environmental impact assessment software such as OpenLCA. A computer-based approach to the method could facilitate its practical application. Future research plans to expand the method to include material analyses across the entire product lifecycle. It is also planned to ensure that a greater number of material combinations for all product criteria are included. Tests of the method will also be conducted on products from various industries. Also, a sensitivity analysis of the method is planned as part of future research.

## 5. Conclusions

Offering product innovations requires reliance on material changes in these products. These changes often involve improving product quality, as it remains the primary reference point for meeting customer requirements. However, sustainable product development requires focusing the product development process on considering other aspects, such as environmental impact throughout the life cycle and manufacturing costs. However, selecting materials is an extremely challenging task. It has been observed that there is no comprehensive method that allows for considering all of these aspects simultaneously. Therefore, the aim of this research was to develop a method for selecting materials for improving the final product while simultaneously considering quality, environmental impact throughout the life cycle, and manufacturing costs.

The test and method illustrations were conducted for a six-speed manual gearbox used in light passenger cars. Three key product criteria were analyzed: the housing, the gear, and the synchronizer ring. Various material modifications were sought, considering materials such as aluminum, cast iron, bronze, magnesium alloy, chrome steel, and others for these criteria. Five main material modifications were created, representing so-called gearbox prototypes. These were assessed for quality (customer satisfaction) using a formal scoring method. These materials were then assessed for their environmental impact, focusing on the material sourcing and extraction stages within the product life cycle. OpenLCA software with the Ecoinvent database was used for this purpose. Subsequently, the obtained results were aggregated and combined in a cost analysis with the actual production costs of the proposed prototypes. Based on the resulting prototype rankings, the most favorable material alternative for the three gearbox criteria analyzed was concluded. In the case under consideration, this was the P1 prototype, which proposed the use of an aluminum housing, a low-alloy steel gear, and a bronze synchronizer ring. This prototype meets the expected quality requirements, has a relatively low negative environmental impact in the first stage of the life cycle, and is therefore cost-effective in terms of production costs. However, decisions regarding the direction of product development are made individually, for example, based on the company’s predispositions, access to materials, or resources.

The results showed that decision-making regarding prototype selection from among possible variants varies. When traditionally considering only quality, the choice differed from that when considering environmentally friendly and other materials, and a cost analysis was also performed. The proposed method can be used for any newly created or modified product. The proposed method is not restricted by industry or product type. However, the method was developed for classic electrical engineering products. Additional assumptions or constraints may need to be introduced for other specific products. To date, the methodology has not been tested in a specific workplace. However, it is planned to test it as part of future research. However, broader validation across multiple industries and product types is still required to broadly apply the method to complex industry cases.

## Figures and Tables

**Table 1:** Literature review on the classification of product materials.

Source	Classification	Main Application
[37]	Classification by material group, i.e., metals, ceramics, polymers, and composites, i.e., by the dominant bond type.	Technical sciences, the process of selecting materials for product design, e.g., for the integration of material substances.
[43]	A classification according to the multiplicity and visual representation of materials that integrates their aesthetic and perceived attributes.	Support in the design of products where visual impact is important.
[37,44,45]	Classification depends on the measured properties, leading to the specificity of the final product in which the raw materials will be contained.	Support in the final design of final products, taking into account, for example, hardness, strength, flexibility, plasticity, ductility, thermal and electrical conductivity, and biodegradability.
[37]	Classification of materials, where material substances are distinguished into raw materials (e.g., primary, secondary, and others) and materials (e.g., ceramics, glass, leather, wood, and others), and these in turn into processed, unprocessed, and other materials.	The process of selecting materials for product design.
[46]	Material classification, including textiles/leather, metal, plastic, composites, elastomer/rubber, wood, ceramics/stone, glass, and others.	Support in the initial stages of product design and beyond.

**Table 2:** Interpretation of the quality-environment-cost (QEC) index value according to the scale of relative states.

Rank of QEC Value	Interpretation of Making Decision
<1.00; 0.95>	Excellent
(0.95; 0.85>	Distinctive
(0.85; 0.75>	Beneficial
(0.75; 0.65>	Satisfactory
(0.65; 0.55>	Moderate
(0.55; 0.45>	Sufficient
(0.45; 0.35>	Unsatisfactory
(0.35; 0.25>	Unfavourable
(0.25; 0.15>	Critical
(0.15; 0.05>	Bad

Source: own elaboration.

**Table 3:** Manual gearbox prototypes with material alternatives for housing, gear, and synchronizer.

Prototypes	Material Alternatives	Expected Benefits	Expected Limitations
P1	housing: aluminum, gear: low-alloy steel, synchronizer ring: bronze	lightweight housing with good thermal conductivity, durable and wear-resistant gear, good friction properties, and durability of the synchronizer	the housing may be less rigid, which affects the noise and vibration levels; hardened steel generates costs for heat treatment and dimensional precision; bronze is susceptible to wear under high loads
P2	housing: cast iron, gear: hardened steel, synchronizer ring: chrome steel	housing that dampens vibrations well and withstands loads, durable gears, and a synchronizer that reduces surface wear	heavy housing, steel heating processes generate costs, relatively higher abrasion of coatings necessitating their quality control and possible re-application
P3	housing: composite with metal reinforcements, gear: low-alloy steel, synchronizer ring: chrome steel	lightweight composite housing, durable gears, durable and abrasion-resistant synchronizers	low thermal conductivity and hardness of the housing, which additionally requires metal inserts and an advanced cooling system; chrome steel synchronizer allows for reducing the friction coefficient, causing synchronization instability
P4	housing: magnesium alloy (or aluminum), gear: hardened steel, synchronizer ring: bronze	lightweight housing, durable gears, synchronizer that reduces surface wear	expensive housing, less resistant to corrosion, requiring anti-corrosion protection
P5	housing: aluminum, gears: low-alloy steel, synchronizer ring: copper–bronze (brass with manganese)	lightweight housing, durable gears, highly resistant synchronizers with improved abrasion resistance	the housing may be less rigid, which affects the noise and vibration levels; hardened steel generates costs for heat treatment and dimensional precision; bronze is susceptible to wear under high loads

**Table 4:** Qualitative assessment of material modifications of selected gearbox criteria.

Prototypes	Case	Sprocket	Synchronizer (Ring)
P1	4	4	3
P2	1	4	3
P3	2	4	4
P4	3	4	3
P5	4	4	4

**Table 5:** Results of qualitative assessment of material modification of selected gearbox criteria according to the formalized scoring method.

Prototypes	P1	P2	P3	P4	P5
P	15.00	12.00	13.00	12.00	18.00
G	0.63	0.50	0.54	0.50	0.75
K	0.00	0.02	0.01	0.00	0.00
Q	0.58	0.47	0.50	0.45	0.70
Ranking	2	4	3	5	1

**Table 6:** Rankings of gearbox prototypes for material modifications of selected criteria depending on aggregated quality–environmental (QE) indicators.

Prototypes	P1	P2	P3	P4	P5
QE	0.68	0.74	0.57	0.23	0.66
Ranking	2	1	4	5	3

**Table 7:** Results of cost analysis.

Analysis	P1	P2	P3	P4	P5
QE	0.68	0.74	0.57	0.23	0.66
QE (%)	68.19	73.50	56.91	22.50	66.04
C (Euro)	276.74	325.58	279.07	281.40	302.33
C_K_	4.06	4.43	4.90	12.51	4.58
k	1.00	0.00	0.95	0.90	0.48
E	1.47	0.00	1.67	4.02	0.72
d	0.66	0.00	0.70	2.01	0.36
c	1.00	0.96	0.90	0.00	0.94
R_t_	0.74	0.52	0.67	0.72	0.61
R_e_	0.93	0.30	0.88	0.77	0.60
QEC	0.84	0.41	0.78	0.74	0.60
Ranking	1	5	2	3	4

## Data Availability

The original contributions presented in this study are included in the article. Further inquiries can be directed to the corresponding author.
